# Intention to Receive the TAK-003 Dengue Vaccine and Associated Factors Among Adults in Rural Northern Thailand

**DOI:** 10.3390/vaccines14050416

**Published:** 2026-05-05

**Authors:** Chanachai Polpitakchai, Sipang Pangprasertkul, Pimbucha Rusmevichientong, Ranchana Yamsiri, Jinjuta Panumasvivat, Wachiranun Sirikul, Ratana Sapbamrer, Sitong Luo, Chunqing Lin, Amornphat Kitro

**Affiliations:** 1 Department of Community Medicine, Faculty of Medicine, Chiang Mai University, Chiang Mai 50200, Thailand; 2 Division of Infectious Diseases, Department of Pediatrics, Faculty of Medicine, Chiang Mai University, Chiang Mai 50200, Thailand; 3 Department of Public Health, California State University Fullerton, Fullerton, CA 92831, USA; 4 Environmental and Occupational Medicine Excellence Center (EnOMEC), Faculty of Medicine, Chiang Mai University, Chiang Mai 50200, Thailand; 5 Department of Biomedical Informatic and Clinical Epidemiology, Faculty of Medicine, Chiang Mai University, Chiang Mai 50200, Thailand; 6 Vanke School of Public Health, Tsinghua University, Beijing 100084, China; 7 Institute for Healthy China, Tsinghua University, Beijing 100084, China; 8 Department of Psychiatry and Biobehavioral Sciences, Semel Institute for Neuroscience and Human Behavior, University of California, Los Angeles, CA 90024, USA

**Keywords:** dengue vaccine, acceptance, rural, Thailand, health equity

## Abstract

**Background**: Dengue is a common mosquito-borne disease in Thailand and poses an increased risk of severe illness among adults. The TAK-003 quadrivalent dengue vaccine is newly introduced in Thailand and has demonstrated promising efficacy. This study aimed to assess intention to receive the vaccine, knowledge, attitudes, and associated factors with TAK-003 among participants. **Methods**: A cross-sectional study was conducted from September 2024 to July 2025 among Thai adults aged ≥20 years living in rural areas of Chiang Mai. Individuals with prior dengue vaccination were excluded. Binary logistic regression identified factors associated with intention to the receive vaccine. **Results**: A total of 482 participants were enrolled. The mean age was 61.3 years (SD 11.5), and 73.2% (n = 353) were female. Most participants had primary education or lower (64.3%, n = 310), and 62.9% (n = 303) reported a monthly household income < 10,000 THB (314 USD). The intention to receive the TAK-003 vaccine was 68.7% (n = 331). Only 31.5–35.1% correctly answered dengue treatment questions, and 38.6% believed they would contract dengue within five years. Concerns regarding vaccine side effects (76.1%) and efficacy (56.4%) were common. Local healthcare providers were the most trusted source of vaccine information (78.0%), followed by doctors (49.2%). Prior influenza vaccination (aOR 1.57, 95% CI 1.02–2.41) and more positive attitudes toward dengue vaccination (aOR 1.06, 95% CI 1.02–1.10) were associated with intention to receive the vaccination. **Conclusions**: Intention to vaccinate with the TAK-003 vaccine among adults in rural Chiang Mai was moderate. These findings can inform community-based vaccination programs and strategic planning for dengue vaccine rollout in rural northern Thailand.

## 1. Introduction

Dengue viral infection is a common mosquito-borne viral disease that is found in more than 100 countries worldwide, especially in tropical and subtropical countries, with up to 400 million people at risk worldwide [1]. Moreover, global warming with higher temperatures, heavy rainfall, and high humidity has triggered dengue outbreaks in new regions where they have never been found [2]. The World Health Organization (WHO) reported a rise in global cases from 505,430 in 2000 to 14.6 million in 2024, with over 12,000 deaths [1]. The annual global costs for treatment were estimated to be 8.9 billion USD (3.7 to 19.7 billion USD) [3]. In 2005, Thailand’s projected national annual cost of dengue was estimated to be approximately 158 million USD [4]. In 2023 alone, Thailand reported 158,620 dengue cases and 181 deaths, with a case fatality rate of 0.11%. Cases were 2.2 times higher, and mortality was 11.7 times higher than in 2020 [5]. Moreover, older adult individuals reported higher rates of severe dengue, prolonged hospitalization, more frequent complications, and a higher mortality rate compared to non-elderly individuals [6].

Thailand has recommended dengue vaccination as a complementary preventive strategy to provide protection and prevent complications from dengue infection [7]. Dengue vaccine CYD-TDV (Dengvaxia) was introduced in 2017, with 65.6% efficacy in preventing dengue, 93.2% efficacy in reducing disease severity, and 80.8% efficacy in reducing hospitalization [7]. This vaccine is approved for ages 6 to 45 years old with a history of previous dengue infection [8]. In 2023, a new dengue vaccine, TAK-003 (Qdenga), was introduced. It is administered as a two-dose regimen, given subcutaneously with a three-month interval, and offers 80.2% efficacy in preventing dengue and 90.4% efficacy in reducing hospitalization. This vaccine is approved for individuals aged above 4 years without requiring prior dengue infection and is currently the primary dengue vaccine recommended in Thailand [9,10,11].

A systematic review and meta-analysis from the United States and Asian countries found that the acceptance rate of the CYD-TDV vaccine was 40–88.4% [12,13], with the highest number, 83.3%, found in Malaysia in 2018 [14]. It is found that the vaccine acceptance rate and positive attitudes towards vaccine acceptance correlated with knowledge about dengue infection among the population [14,15]. Individuals who were concerned about themselves or their children being at a higher risk of getting the infection in the next five years were around two times more likely to accept the vaccine. Additionally, vaccine acceptance among individuals diagnosed with dengue infection was two times higher compared to those who had not been diagnosed [16]. Further studies in Malaysia and Brazil in 2022 and 2023 showed that individuals with knowledge of the disease, symptoms, prevention methods, and trust in the benefits of the vaccine tend to have a good attitude toward vaccine acceptance [17,18,19]. In 2018, a qualitative study in the Philippines found that the vaccine acceptance rate was increased by trust in the healthcare system, knowledge about the vaccine, and the previous dengue infection experience of parents [20]. Furthermore, a study in Puerto Rico in 2023 found that individuals who had received the influenza vaccine in the previous year were more likely to accept the dengue vaccine [21].

Information regarding the intention to vaccinate with the newly launched dengue vaccine among adults in rural Thailand, where disparities in health knowledge and access to vaccination remain pronounced, is still limited. This study aims to determine the intention to vaccinate with the TAK-003 dengue vaccine, as well as the knowledge, attitudes, and factors influencing its uptake among this underserved population. The findings will help inform the development of tailored strategies and communication approaches to enhance vaccine acceptance and reduce health disparities among adults living in rural Thailand. By generating context-specific evidence, this research can guide physicians and healthcare workers in creating practical recommendations for dengue prevention and control. Moreover, the findings can support local health initiatives to improve vaccine confidence, promote equitable access to immunization, and strengthen dengue prevention interventions, particularly for populations in resource-limited setting.

## 2. Materials and Methods

### 2.1. Setting and Study Design

This cross-sectional study was conducted in Chiang Mai, Thailand, between September 2024 and July 2025. Participants included Thai individuals aged 20 years and above residing in rural areas of Chiang Mai, Thailand. Chiang Mai is the second-largest province in Thailand and consistently ranks among the top five provinces with the highest reported dengue cases [22]. Participants who had received a prior dengue vaccine were excluded. Data were collected using a structured questionnaire distributed in collaboration with subdistrict health promotion hospitals in Chiang Mai and the village health volunteers, who facilitated community outreach and household visits.

### 2.2. Participants and Recruitment

The research team promoted the study through partner subdistrict health promotion hospitals in Chiang Mai. Trained research staff, with support from village health volunteers, conducted community visits in rural areas and personally invited eligible residents who were at home during weekdays to participate. A convenience sampling approach was used. Data were completed either on paper or on tablets provided by the research team, with staff assistance available when needed, for example to clarify terms or questions. The response rate for complete questionnaires was 89.1%, with 59 participants excluded due to incomplete responses.

### 2.3. Sample Size Calculation

The sample size was calculated for estimating an infinite population proportion. The proportion of individuals was assumed to be 0.883 [12], with an error margin (d) of 0.05, an alpha level of 0.01, and a Z (0.995) value of 2.58. Cluster sampling was not applied. As a result, the required sample size was 276 participants, and accounting for a 20% dropout rate, a total of 332 participants were needed.

### 2.4. Questionnaire

The study utilized a questionnaire divided into four sections.

1. Sociodemographic data, including age, gender, occupation, education level, monthly income, number of household members, history of immunocompromised condition, history of influenza vaccination in the past year (yes/no), and history of dengue viral infection (yes/no), intention to receive the new dengue vaccine (yes/no).
2. Knowledge about dengue disease, including disease transmission, symptoms, treatment, and vaccine availability for ten items. Each of the 10 knowledge items were scored as 1 for a correct response and 0 for an incorrect response, resulting in a total possible score ranging from 0 to 10, with scores of greater than 7 indicating good knowledge.
3. Attitudes toward dengue disease and the dengue vaccine: Participants’ attitudes toward dengue fever and the dengue vaccine were assessed using a five-level Likert scale, ranging from the lowest degree (1) to the highest degree (5) for ten items. Each of the 10 attitude items was measured on a 5-point Likert scale (1 = strongly disagree to 5 = strongly agree), yielding a total score ranging from 10 to 50, with scores greater than 38 indicating positive attitudes toward dengue viral infection and vaccination.
4. Concerns and trusted sources of information regarding dengue vaccine decision-making (multiple responses allowed).

To ensure validity, the questionnaire was reviewed by experts, including pediatric infectious disease specialists, adult infectious disease specialists, and physicians specializing in vaccines and tropical infectious diseases. The reliability of the structured questionnaire was assessed for its two distinct sections: the ten-item knowledge and the ten-item attitude. For the ten knowledge items, which were scored dichotomously, the Kuder–Richardson 20 (KR-20) coefficient was calculated to assess reliability. The analysis yielded a KR-20 coefficient of 0.387 while item difficulty was around 0.6. For the ten attitude items, which were measured on a Likert scale, Cronbach’s alpha was calculated to assess internal consistency. The analysis demonstrated a Cronbach’s alpha coefficient of 0.806. We also performed a 1000-sample bootstrap analysis to confirm the stability of Cronbach’s alpha coefficient. The results yielded a 95% confidence interval for Cronbach’s alpha ranging from 0.769 to 0.843. In addition, the item-total correlations and the corrected item-total correlations between 0.20 and 0.80 were considered acceptable [23].

### 2.5. Statistical Analysis

All statistical analyses were conducted using STATA statistical software (StataCorp, 2023; Stata Statistical Software: Release 18; StataCorp LLC, College Station, TX, USA). Descriptive statistics were used to summarize sociodemographic characteristics, history of dengue infection, and vaccine-related attitudes and knowledge. The normality of continuous variables was assessed using the Shapiro–Wilk test, supplemented by the visual inspection of histograms. Continuous variables with normal distribution were reported as mean and standard deviation (SD) and were compared between groups using an independent *t*-test, while non-normally distributed variables were summarized as median and interquartile ranges (IQR). Categorical variables were summarized as frequencies and percentages and analyzed using Fisher’s exact test. A univariable analysis was initially performed to identify potential factors associated with the dependent variables. Variables with a *p*-value < 0.2 in the univariable analysis were subsequently included in the multivariable models. Binary logistic regression analysis was performed to determine factors associated with intention to vaccinate with the dengue vaccine. Adjusted odds ratios (aORs) and 95% confidence intervals (95% CI) were estimated using the enter method. The enter method is a non-stepwise approach in which all selected independent variables are entered into the model simultaneously, without automatic selection or removal based on statistical criteria. Independent variables included in the binary logistic model were age, education levels, history of flu vaccination, history of refuse vaccine, total scores of knowledges, and total scores of attitudes. All tests were two-tailed, and a *p*-value < 0.05 was considered statistically significant.

## 3. Results

A total of 482 participants were recruited and included in the analysis. Overall, 68.7% (95% CI: 64.3–72.8%, n = 331) reported intention to receive the dengue vaccine. The mean age was 61.3 years (SD = 11.5), and 73.2% (n = 353) were female. Most participants (64.3%, n = 310) had a primary education or lower, 40.7% (n = 196) were employed, and 62.7% (n = 302) were married. Additionally, 62.9% (n = 303) reported a monthly family income of less than 10,000 THB (314 USD). Regarding health status and dengue-related history, 3.5% (n = 17) had an immunocompromised condition, 10.2% (n = 49) had a previous dengue infection, 7.9% (n = 38) had been hospitalized for dengue, 17.2% (n = 83) had a family history of dengue, and 32.6% (n = 157) knew a friend or acquaintance who had been diagnosed with dengue. Moreover, 48.1% (n = 232) had received an influenza vaccine in the past year, while 13.7% (n = 66) reported past vaccine refusal (Table 1).

Regarding dengue-related knowledge, 96.9% (n = 467) correctly identified that dengue is transmitted by Aedes mosquitoes. However, only 49.8% (n = 240) were aware that Aedes mosquitoes primarily bite during the daytime, and 82.2% (n = 396) recognized common breeding sites. Additionally, 79.7% (n = 384) understood that a person can contract dengue more than once in their lifetime. With respect to symptoms, 69.7% (n = 336) correctly identified common dengue symptoms. Only 31.5% (n = 152) knew that not all dengue patients require a blood transfusion, and 35.1% (n = 169) were aware that there is no specific antiviral medication for dengue. Finally, 61.2% (n = 295) correctly identified that a dengue vaccine is available in Thailand (Table 2).

Regarding perceptions and attitudes toward dengue infection, 88.8% (n = 428) agreed or strongly agreed that dengue is a severe disease for children, while 75.7% (n = 365) felt that it is also a serious disease for adults. In terms of personal risk perception, 38.6% (n = 186) agreed or strongly agreed that they are likely to contract dengue within the next five years, and 43.4% (n = 209) believed that their children or grandchildren may also be at risk during the same period. Attitudes toward vaccination were generally positive. A majority (75.5%, n = 364) agreed or strongly agreed that the benefits of the dengue vaccine outweigh its side effects. Additionally, 73.0% (n = 352) believed that the vaccine helps prevent dengue infection, 81.1% (n = 391) agreed or strongly agreed that vaccination reduces hospitalization, and 85.1% (n = 410) agreed or strongly agreed that it helps reduce dengue-related mortality (Table 3).

Regarding vaccine concerns, 76.1% (n = 367) expressed concerns about side effects, followed by 56.4% (n = 272) who were worried about vaccine efficacy. Additionally, 55.4% (n = 267) felt that the vaccine was still too new, and 38.0% (n = 183) reported concerns about potential long-term effects. Regarding trusted sources of information influencing dengue vaccination decisions among participants, most (78.0%, n = 376) obtained information from local healthcare providers, including community nurses, public health officers, and village health volunteers, while 49.2% (n = 237) received information from doctors. Other sources included television (38.8%, n = 187) and the internet (32.0%, n = 154) (Table 4).

Factors significantly associated with the intention to vaccinate with the dengue vaccine included having a secondary education, which increased the likelihood of receiving the vaccine by 2.1 times compared with those with primary education or lower (aOR = 2.104, 95% CI: 1.205–3.674, *p* < 0.05). Participants who had received an influenza vaccine in the past year were also 1.6 times more likely to intend to receive the dengue vaccine (aOR = 1.571, 95% CI: 1.023–2.411, *p* < 0.05). Additionally, higher overall attitude scores were associated with an intention to vaccinate (aOR = 1.062, 95% CI: 1.024–1.101, *p* < 0.01). Participants who perceived the vaccine as safe were more likely to intend to vaccinate compared with those who did not perceive it as safe (aOR 1.279; 95% CI: 1.024–1.596, *p* < 0.05) (Table S1). In contrast, those with a history of vaccine refusal were 62% less likely to intend to receive the dengue vaccine (aOR = 0.379, 95% CI: 0.216–0.663, *p* < 0.01) (Table 5).

## 4. Discussion

This study provides early evidence of the intention to receive the TAK-003 dengue vaccine among the study participants living in rural Chiang Mai, Thailand, which was 68.7%. Factors positively associated with the intention to vaccinate with the new dengue vaccine included secondary education, prior influenza vaccination, and positive attitudes toward dengue vaccination, while prior vaccine refusal was negatively associated.

The intention to vaccinate observed in this study is lower than rates reported in countries with a high dengue burden, such as Malaysia (88.4%) and the Philippines (95.5%), where dengue vaccination programs have been previously introduced. It is also lower than the acceptance rate observed in a quadrivalent dengue vaccine study conducted in Brazil (87%) [14,24,25]. This may be related to differences in perceived risk, as only 38.6% of adults in rural areas perceived themselves at high risk of dengue in the future. Moreover, the survey was conducted one year after Thailand’s major dengue outbreak in 2023, during which awareness could have been lower [5]. Our findings further showed that individuals with a more positive attitude toward vaccination were 10% more likely to receive the dengue vaccine, especially those who perceived the vaccine as safe. These results highlight the need to address gaps in awareness and perceptions to support dengue prevention in the study population. Targeted health promotion should emphasize that dengue risk persists even during off-peak seasons and that vaccination remains a safe and effective means of primary prevention in endemic areas. Socioeconomic disparities, including lower education and income, may be associated with inequitable vaccine uptake in rural settings. Therefore, local health providers could promote equitable access by improving vaccine availability and implementing targeted educational programs to enhance knowledge and awareness of dengue prevention and vaccination.

A common misconception among adults living in rural areas in our study was that dengue has a specific cure and often requires a blood transfusion; only one-third of participants answered these questions correctly. Similar findings were observed in an Indonesian study, where about 70% of respondents believed dengue could be cured, and in a systematic review across Asian populations, where approximately 75% were uncertain about the availability of a cure [26]. Despite dengue being a common public health concern in tropical countries like Thailand, misconceptions remain widespread, particularly among adults in rural areas, suggesting potential disparities in health literacy and access to reliable information. Local healthcare providers can play a crucial role in bridging this gap by delivering accurate, accessible, and culturally appropriate education to this population [27]. Given the absence of specific antiviral treatment and the higher risk of severe outcomes among adults, addressing these misconceptions may be important [6]. Strengthening community knowledge on disease management, promoting mosquito bite prevention, eliminating breeding sites, and raising awareness of dengue severity may help reduce misinformation, improve preventive behaviors, and ultimately lessen the disease burden in underserved communities.

Interestingly, this study found that 78% of participants reported local healthcare providers such as community nurses, public health officers, and village health volunteers who work in Subdistrict Health Promotion Hospital as trusted sources of information informing their decisions about dengue vaccination. In comparison, doctors (50%) and the television (39%) ranked second and third, respectively. This pattern differs from findings in a previous study on COVID-19 vaccine acceptance in Thailand, where 82% of participants reported doctors as their most trusted source of vaccine information, as well as studies from rural areas of the southern United States, where medical providers were also regarded as the most trusted messengers [28]. These results suggest the important role of community-based healthcare providers who work closely with local populations, particularly adults in rural areas, where accessibility and familiarity may be associated with greater trust than professional authority alone [29]. This finding suggests a potential need for community-led communication strategies by empowering community primary care staff as key messengers, while involving doctors as team members to provide updated information on vaccine safety, efficacy, and other concerns. Such collaborative, trust-based strategies may help reduce vaccine hesitancy, increase vaccine uptake, and sustain overall community health, particularly in underserved populations.

Our study found that participants who received the influenza vaccine in the past year were 1.6 times more likely to intend to receive the dengue vaccine. This aligns with prior studies among Thai healthcare workers and individuals with chronic conditions, where those who had received a flu shot were about twice as likely to accept a COVID-19 booster, as well as findings from global post-COVID-19 trends in which individuals familiar with routine vaccinations demonstrated greater openness to new vaccines [21,30,31]. This trend may indicate that individuals with greater health awareness, particularly regarding the risks of infection, complications, hospitalization, and healthcare costs, are more inclined to accept additional vaccines [21]. For adults who regularly receive free seasonal influenza vaccination under the Thai government program, healthcare providers could use these visits as opportunities to raise awareness about the availability, benefits, and risks of the new dengue vaccine, encouraging informed decision-making [14]. Moreover, integrating information on other adult vaccines such as influenza, shingles, pneumococcal, tetanus-diphtheria-pertussis (Tdap), and respiratory syncytial virus (RSV) during these visits may help reduce disparities in vaccine access and awareness, especially among those living in rural areas or with lower educational attainment, groups often shown to experience greater barriers to vaccination [32].

This study has several limitations. Its cross-sectional design prevents causal inference, and the use of self-reported data introduces the possibility of recall biases especially for dengue and vaccination history. Due to convenience sampling in the community, the study sample was predominantly composed of adults aged 50 years and older, as recruitment targeted individuals who were at home during weekdays. This may have introduced selection and social desirability biases, potentially underestimating or overestimating the intention to vaccinate. Additionally, the study was conducted in a single center, which may limit the generalizability of the findings to other regions of Thailand or other countries with differing sociodemographic contexts. The absence of information on personal prevention practices, such as mosquito-bite prevention or mosquito-breeding site elimination, also limits the ability to assess how these behaviors may influence knowledge, attitudes, or intention to receive the new dengue vaccine. The internal consistency of the 10-item knowledge scale was relatively low, indicating limited reliability. The findings related to knowledge should be interpreted with caution as the scale may not fully capture participants’ knowledge levels. Future studies should examine intention to vaccinate under different scenarios, e.g., varying cost, accessibility, previous experiences, campaign exposure, and physician recommendation to better understand and reflect potential uptake in real-world settings. A health equity framework may be applied to better understand the complex associations of health inequities and to inform targeted strategies and interventions that address these factors. Despite these limitations, the findings offer important implications. They can inform the development of tailored strategies to address misconceptions, foster more positive attitudes, and improve communication approaches to enhance intention to vaccinate and reduce health disparities among rural adults. Physicians can play a leading role by collaborating with local healthcare providers to generate context-specific evidence and address concerns regarding the safety and efficacy of the new TAK quadrivalent dengue vaccine. Furthermore, the results support the implementation of community-based vaccination programs to strengthen vaccine confidence, promote informed decision making, and advance dengue prevention intervention efforts to increase vaccine uptake, particularly among populations in resource-limited settings.

## 5. Conclusions

This study highlights that approximately two-thirds of adults in rural northern Thailand expressed an intention to receive the TAK-003 dengue vaccine. Around 61.2% were aware that a dengue vaccine is available in Thailand. Prior influenza vaccination and positive attitudes toward dengue vaccination were associated with higher intention to vaccinate with the dengue vaccine, whereas a history of vaccine refusal was negatively associated. Public health efforts should focus on strengthening vaccine confidence by using trusted communication channels and addressing misconceptions or concerns related to vaccine safety and efficacy. The insights from this study can support local health initiatives in strategic planning for dengue vaccine interventions, particularly by promoting equitable access to immunization and strengthening dengue prevention efforts to reduce barriers and improve health equity. These findings may also inform educational initiatives in Thailand and other dengue-endemic settings with similar population characteristics.

## Figures and Tables

**Table 1:** Sociodemographic Characteristics of the Study Participants Living in Rural Areas of Chiang Mai, Thailand.

Parameters	Total	Intention to Receive Vaccine		*p* Value
		Yes (n = 331)	No (n = 151)	
Mean age (SD)	61.3(11.5)	60.1(11.5)	64.1(10.9)	<0.001 **^a^
Sex				
Male	129 (26.8)	91 (27.5)	38 (25.2)	0.658 ^b^
Female	353 (73.2)	240 (72.5)	113 (74.8)	
Education				
Primary or lower	310 (64.3)	197 (59.5)	113 (74.8)	0.002 **^b^
Secondary	138 (28.6)	111 (33.5)	27 (17.9)	
Bachelor or above	34 (7.1)	23 (6.9)	11 (7.3)	
Occupation				
Farmers	57 (11.8)	37 (11.2)	20 (13.2)	0.778 ^b^
Employees	196 (40.7)	136 (41.1)	60 (39.7)	
Own business	97 (20.1)	68 (20.5)	29 (19.2)	
No job/retired	92 (19.1)	60 (18.1)	32 (21.2)	
Others	40 (8.3)	30 (9.1)	10 (6.6)	
Status				
Single	64 (13.3)	44 (13.3)	20 (13.2)	0.550 ^b^
Married	302 (62.7)	212 (64.0)	90 (59.6)	
Divorced/widowed	116 (24.1)	75 (22.7)	41 (27.2)	
Average number of family members	3.4 ± 1.6	3.4 ± 1.6	3.2 ± 1.6	0.488 ^a^
Average number of family members < 18 years old	0.6 ± 0.8	0.6 ± 0.9	0.5 ± 0.8	0.540 ^a^
Family monthly income (THB)				
<5000 (<157 USD)	149 (30.9)	95 (28.7)	54 (35.8)	0.335 ^b^
5001–10,000 (157–314 USD)	154 (32.0)	105 (31.7)	49 (32.5)	
10,001–20,000 (315–623 USD)	98 (20.3)	70 (21.1)	28 (18.5)	
20,001–30,000 (624–942 USD)	49 (10.2)	35 (10.6)	14 (9.3)	
>30,001 (>942 USD)	32 (6.6)	26 (7.9)	6 (4.0)	
Family type				
Single	329 (68.3)	225 (68.0)	104 (68.9)	0.916 ^b^
Complex	153 (31.7)	106 (32.0)	47 (31.1)	
Immunocompromised condition	17 (3.5)	14 (4.2)	3 (2.0)	0.291 ^b^
History of dengue diagnosed	49 (10.2)	37 (11.2)	12 (7.9)	0.331 ^b^
History of dengue hospitalized	38 (7.9)	26 (7.9)	12 (7.9)	0.972 ^b^
History of family dengue diagnosed	83 (17.2)	59 (17.8)	24 (15.9)	0.697 ^b^
Friend/acquaintance diagnosed	157 (32.6)	113 (34.1)	44 (29.1)	0.296 ^b^
History of flu vaccination in the past year	232 (48.1)	167 (50.5)	65 (43.0)	0.141 ^b^
History of refuse vaccine	66 (13.7)	30 (9.1)	36 (23.8)	<0.001 **^b^
Total scores of knowledges	6.77 ± 1.62	6.88 ± 1.53	6.52 ± 1.78	0.025 *^a^
Scores ≤ 7	319 (66.2)	209 (63.1)	110 (72.8)	0.023 *^b^
Scores > 7	163 (33.8)	122 (36.9)	41 (27.2)	
Total scores of attitudes	38.23 ± 5.80	38.89 ± 5.72	36.76 ± 5.71	<0.001 **^a^
Scores ≤ 38	259 (53.7)	166 (50.2)	93 (61.6)	0.012 *^b^
Scores > 38	223 (46.3)	165 (49.8)	58 (38.4)	

^a^ analyzed with independent *t*-test; ^b^ analyzed with Fisher exact test. * *p*-value < 0.05, ** *p*-value < 0.001.

**Table 2:** Knowledge About Dengue and Vaccination Among the Study Participants Living in Rural Areas of Chiang Mai, Thailand.

Statement	Answer Correctly (n, %)
1. Dengue disease is transmitted by Aedes mosquitoes (Y)	467 (96.9)
2. Aedes mosquitoes bite during the daytime (Y)	240 (49.8)
3. Aedes mosquitoes breed and lay eggs in stagnant, clear water (Y)	396 (82.2)
4. One person can contract dengue disease more than once in a lifetime (Y)	384 (79.7)
5. Children are more prone to contracting dengue fever (N)	363 (75.3)
6. Fever, nausea, and vomiting can be found in dengue fever (Y)	336 (69.7)
7. Dengue fever can be fatal (Y)	460 (95.4)
8. Every person with dengue fever requires a blood transfusion (N)	152 (31.5)
9. There are specific medicines that can cure dengue disease (N)	169 (35.1)
10. A dengue vaccine is available in Thailand (Y)	295 (61.2)

**Table 3:** Perceptions and Attitudes Toward Dengue and Vaccination Among the Study Participants Living in Rural Areas of Chiang Mai, Thailand.

Statement	Strongly Disagree	Disagree	Unsure	Agree	Strongly Agree
1. I believe dengue fever is a serious disease for children	8 (1.7)	21 (4.4)	25 (5.2)	212 (44.0)	216 (44.8)
2. I believe dengue fever is a serious disease for adults	14 (2.9)	43 (8.9)	60 (12.4)	229 (47.5)	136 (28.2)
3. I think I am at risk of contracting dengue fever in the next 5 years	47 (9.8)	115 (23.9)	134 (27.8)	147 (30.5)	39 (8.1)
4. I think my child/grandchild is at risk of contracting dengue fever in the next 5 years	48 (10.0)	89 (18.5)	136 (28.2)	158 (32.8)	51 (10.6)
5. I believe the dengue vaccine has been thoroughly tested for safety	9 (1.9)	32 (6.6)	103 (21.4)	216 (44.8)	122 (25.3)
6. I believe the dengue vaccine can help boost the immune system in both children and adults	5 (1.0)	26 (5.4)	58 (12.0)	237 (49.2)	156 (32.4)
7. The benefits of the vaccine outweigh the side effects	7 (1.5)	22 (4.6)	89 (18.5)	226 (46.9)	138 (28.6)
8. I believe the vaccine helps prevent dengue infection	19 (3.9)	38 (7.9)	73 (15.1)	226 (46.9)	126 (26.1)
9. I believe the vaccine can reduces the likelihood of hospitalization due to dengue	6 (1.2)	29 (6.0)	56 (11.6)	254 (52.7)	137 (28.4)
10. I believe the vaccine can help reduce deaths from dengue fever	5 (1.0)	13 (2.7)	54 (11.2)	238 (49.4)	172 (35.7)

**Table 4:** Vaccine Concerns and Trusted Sources of Information on Dengue Vaccination Among Adults Living in Rural Areas of Chiang Mai, Thailand.

Parameter	(n, %)
Vaccine concern	
1. Side effects/Safety concerns	367 (76.1%)
2. Efficacy concerns	272 (56.4%)
3. Belief that vaccination is premature	267 (55.4%)
4. Long-term effects	183 (38.0%)
Trusted Sources of Information Influencing Dengue Vaccination Decisions	
1. Local healthcare providers *	376 (78.0%)
2. Doctors	237 (49.2%)
3. Television	187 (38.8%)
4. Internet	154 (32.0%)
5. Government sources	137 (28.4%)
6. Friends and family	115 (23.9%)
7. Other sources	75 (15.6%)

* Local Healthcare providers, including community nurses, public health officers, and village health volunteers.

**Table 5:** Binary Logistic Regression for Investigating Factors Associated with Intention to Vaccinate Among Adults Living in Rural Areas of Chiang Mai, Thailand.

Factors	Adjusted OR	95% CI	*p*-Value
Age	0.978	0.956, 1.001	0.065
Education levels (Primary or lower as ref.)			
Secondary	2.104	1.205, 3.674	0.009 *
Bachelor	1.142	0.490, 2.663	0.759
History of flu vaccination	1.571	1.023, 2.411	0.039 *
History of vaccine refusal	0.379	0.216, 0.663	<0.001 **
Total scores of knowledges	1.034	0.909, 1.176	0.610
Total scores of attitudes	1.062	1.024, 1.101	<0.001 **

Analyzed with binary logistic regression analysis with the enter method. * *p*-value < 0.05, ** *p*-value < 0.001.

## Data Availability

The original contributions presented in the study are included in the article/Supplementary Material, further inquiries can be directed to the corresponding author.

## Supplementary Materials

The following supporting information can be downloaded at: https://www.mdpi.com/article/10.3390/vaccines14050416/s1, Table S1: Binary Logistic Regression for investigating Factors Associated with Intention to Vaccination Among Adults Living in Rural Areas of Chiang Mai, Thailand; Table S2: Multicollinearity assessment of predictors in the binary logistic regression model for factors associated with intention to vaccinate among adults in rural Chiang Mai, Thailand.
