# Supplementary material for: Intention to Receive the TAK-003 Dengue Vaccine and Associated Factors Among Adults in Rural Northern Thailand
Source: Vaccines (Basel). 2026 May 5;14(5):416. doi: 10.3390/vaccines14050416 (PMC13211337; doi:10.3390/vaccines14050416)
Supplement: Supplementary file 1 [file vaccines-14-00416-s001.zip › vaccines-4272528-supplementary.pdf]

Table S1. Binary Logistic Regression for investigating Factors Associated with Intention to Vaccination Among Adults Living in Rural Areas of Chiang Mai, Thailand.

Factors	Adjusted OR	95% CI	P-value
Age	0.978	0.956, 1.001	0.065
Education levels (Primary or lower as ref.)			
Secondary	1.926	1.095, 3.387	0.023*
Bachelor	1.218	0.514, 2.885	0.655
History of flu vaccination	1.622	1.038, 2.457	0.027*
History of refuse vaccine	0.375	0.222, 0.685	<0.001**
Total scores of knowledges	0.917	0.732, 1.148	0.448
Attitude: Perceive severity	1.062	1.024, 1.102	0.827
Attitude: Perceive risk	1.048	0.947,1.160	0.361
Attitude: Perceive safety of vaccine	1.279	1.024, 1.596	0.030*
Attitude: Perceive vaccine effectiveness	1.268	0.898, 1.790	0.178

Analyzed with binary logistic regression analysis with the Enter method
*p-value<0.05, **p-value<0.001

Table S2. Multicollinearity assessment of predictors in the binary logistic regression model for factors associated with intention to vaccinate among adults in rural Chiang Mai, Thailand

Determinant	VIF	Square-root VIF
Age	1.49	1.22
Less than primary	(reference category)	
Secondary	1.33	1.15
Bachelor	1.16	1.08
Flu vaccination	1.08	1.04
No vaccination	1.05	1.02
Total attitudes score	1.06	1.03
Total knowledge score	1.09	1.04
